# Proteomics of lung tissue reveals differences in inflammation and alveolar-capillary barrier response between atelectasis and aerated regions

**DOI:** 10.1038/s41598-022-11045-7

**Published:** 2022-04-29

**Authors:** Azman Rashid, Congli Zeng, Gabriel Motta-Ribeiro, Simon T. Dillon, Towia A. Libermann, Marcos Adriano Lessa, Aranya Bagchi, John Hutchinson, Marcos F. Vidal Melo

**Affiliations:** 1 Department of Anesthesia, Critical Care, and Pain Medicine, Massachusetts General Hospital, Boston, MA USA; 2 The University of Queensland, Brisbane, Australia; 3 Department of Anesthesiology, Vagelos College of Physicians and Surgeons, Columbia University, New York, NY USA; 4 Biomedical Engineering Program, Alberto Luiz Coimbra Institute for Graduate Studies and Research in Engineering, Universidade Federal do Rio de Janeiro, Rio de Janeiro, Brazil; 5 BIDMC Genomics, Proteomics, Bioinformatics and Systems Biology Center, Harvard Medical School and Beth Israel Deaconess Medical Center, Boston, MA USA; 6 Department of Biostatistics, Harvard Chan School of Public Health, Boston, MA USA

**Keywords:** Biotechnology, Physiology, Systems biology, Biomarkers, Medical research

## Abstract

Atelectasis is a frequent clinical condition, yet knowledge is limited and controversial on its biological contribution towards lung injury. We assessed the regional proteomics of atelectatic versus normally-aerated lung tissue to test the hypothesis that immune and alveolar-capillary barrier functions are compromised by purely atelectasis and dysregulated by additional systemic inflammation (lipopolysaccharide, LPS). Without LPS, 130 proteins were differentially abundant in atelectasis versus aerated lung, mostly (n = 126) with less abundance together with negatively enriched processes in immune, endothelial and epithelial function, and Hippo signaling pathway. Instead, LPS-exposed atelectasis produced 174 differentially abundant proteins, mostly (n = 108) increased including acute lung injury marker RAGE and chemokine CCL5. Functional analysis indicated enhanced leukocyte processes and negatively enriched cell–matrix adhesion and cell junction assembly with LPS. Additionally, extracellular matrix organization and TGF-β signaling were negatively enriched in atelectasis with decreased adhesive glycoprotein THBS1 regardless of LPS. Concordance of a subset of transcriptomics and proteomics revealed overlap of leukocyte-related gene-protein pairs and processes. Together, proteomics of exclusively atelectasis indicates decreased immune response, which converts into an increased response with LPS. Alveolar-capillary barrier function-related proteomics response is down-regulated in atelectasis irrespective of LPS. Specific proteomics signatures suggest biological mechanistic and therapeutic targets for atelectasis-associated lung injury.

## Introduction

Pulmonary atelectasis affects many surgical patients during perioperative period and critically ill patients and has been frequently cited as a contributing factor for lung injury and acute respiratory distress syndrome (ARDS)^1–3^. While the physiology and clinical relevance of atelectasis are well established, there is surprisingly scant data and controversial information on its biological effects. This limits the pathophysiological assessment of current clinical arguments such as the use of permissive atelectasis versus open lung mechanical ventilation in surgical^1^ and critically ill^4^ patients. It also hinders the understanding of potentially conflicting human findings on the association of regional inflammation and lung expansion. For example, immune response in non-ventilated lung is higher when it kept atelectatic than when exposed to continuous positive pressure in patients during one-lung ventilation undergoing transthoracic oesophagectomy^5^. In contrast, another clinical study with same surgery reported less pronounced inflammatory response in the non-ventilated than ventilated lung^6^. Notably, increased inflammatory cytokine TNF-α in the atelectatic lung during one-lung ventilation has been found correlated with clinical pulmonary outcomes^7^.

Studies addressing cellular immune function and cytokine production in atelectatic lung have reported conflicting enhanced^5,8^ and impaired^9,10^ responses. During atelectasis, the increased inflammatory responses included activation of alveolar macrophages and neutrophils with cytokine release^11,12^; and the infiltration of neutrophils in atelectatic or peri-atelectatic regions^12,13^. Thus, the attenuation of bacterial growth and translocation in experimental model with pneumonia during open lung ventilation is consistent with such processes^14^. Yet, depressed phagocytic activity of alveolar macrophages has also been reported indicating the impairment of immune function during atelectasis^10^. Additionally, reduced blood flow in atelectatic regions could further decrease local load of inflammatory cells and circulating mediators^15^.

Recently, we have reported remarkably distinct of transcriptomics in atelectasis at its early stage, characterized by major lung injury processes with dysregulated immune response and alveolar-capillary barrier function^16^. Systemic endotoxin converted the transcriptomic patterns of atelectasis to increased inflammatory responses, and persistent alveolar-capillary barrier dysfunction. However, proteins are ultimately the functional tissue molecules and potential primary targets for therapeutic intervention. There is substantial distinction between gene expression and corresponding protein concentrations^17^. Accordingly, it is essential to understand the effects of atelectasis on tissue protein levels and associated pathways. An early proteomic study suggested less protein changes in dependent lung regions presumably containing atelectasis than the aerated lung in preterm lambs with short periods of mechanical ventilation^18^. However, no proteomic data specific to atelectatic tissue has been studied. It is currently unknown whether proteomic patterns in atelectasis are consistent with recent genomic reports, and whether there is a specific proteomic signature in atelectatic lung tissues.

In this study, we established lung atelectasis in a large animal model with timeframe of 8 h, relevant to the clinical perioperative period involving surgeries using one-lung ventilation. Based on genomic results, we hypothesize that the proteomic pattern (individual proteins, processes, and pathways) in atelectasis consists of a reduced immune response in the absence of systemic inflammation but an enhanced response in its presence. We further postulate that the proteomic pattern of atelectasis reflects alveolar-capillary barrier compromise, irrespective of systemic inflammation. Our aims are: (1) to compare proteomics response in atelectatic versus aerated lung, with and without systemic inflammation by utilizing a novel proteomic platform; (2) to identify relevant processes and pathways in atelectasis; and (3) to assess the consistency between proteomics and transcriptomics findings.

## Results

### Cardiopulmonary parameters worsened globally and regionally with atelectasis and systemic endotoxin

Cardiopulmonary variables were normal at baseline. Lung compliance and Pa_O2_/F_IO2_ decreased after 8 h of atelectasis. Lipopolysaccharide (LPS) exposure further decreased Pa_O2_/F_IO2_ ratio and cardiac output relative to baseline (Table S1). The ventilated lung had normal regional aeration (0.61 ± 0.08) and strain (0.51 ± 0.34) (Table S2). Without LPS, regional blood volume normalized to lung tissue measured by positron emission tomography (PET) using imaging tracer 18F-fluorodeoxyglucose (FDG) was comparable in atelectatic and aerated lung regions, while it was lower in atelectatic than in aerated lung during LPS exposure (Table S2).

### Proteomics patterns changed in atelectatic versus aerated lung

**Figure 1:**
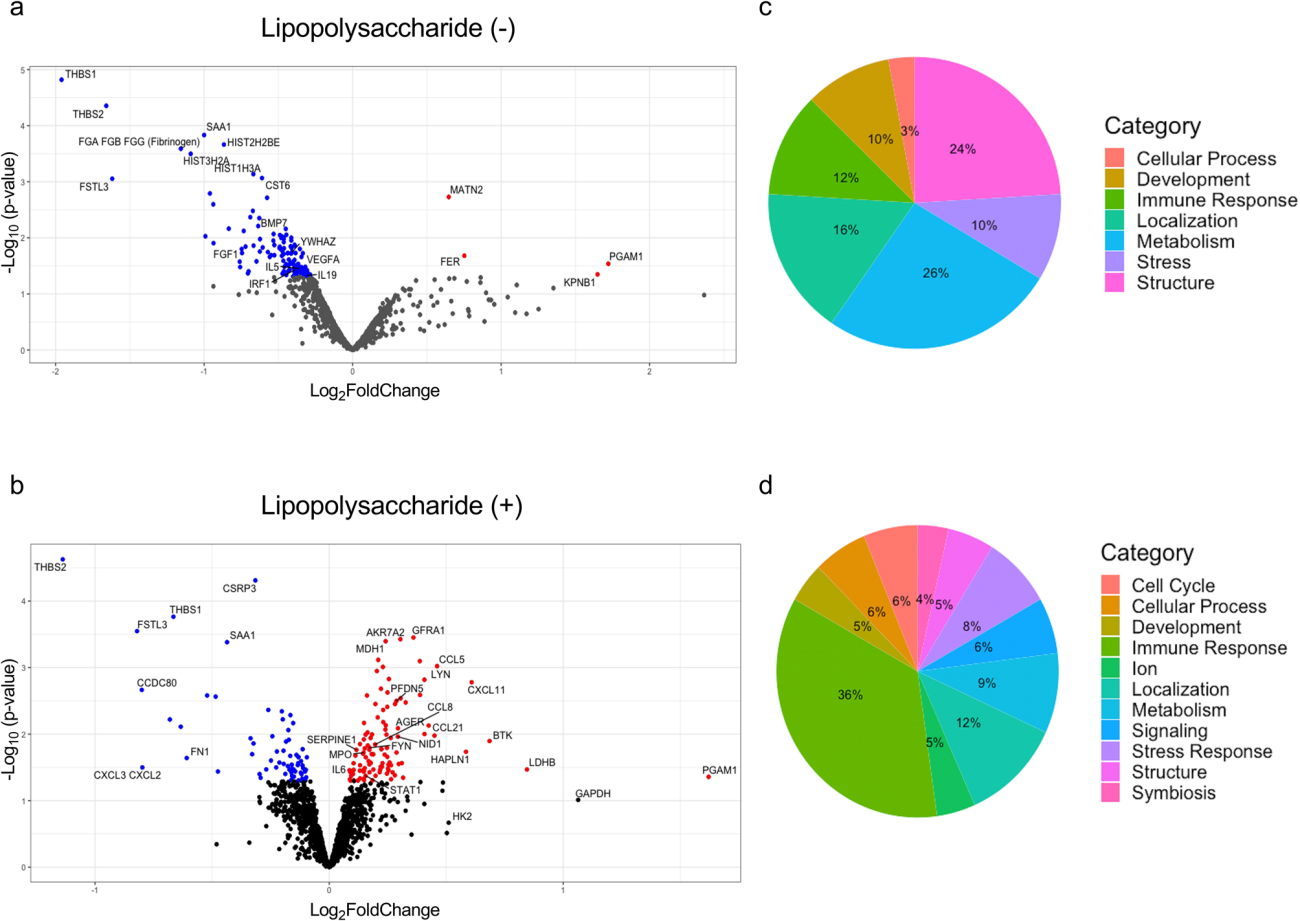
Regional proteomics and processes in the absence or presence of LPS. (**a**) Volcano plot for the condition of LPS(−) showing proteins mostly had less abundance in atelectatic than in aerated lung. (**b**) Volcano plot for the condition of LPS(+) presenting mostly increased proteins in atelectasis. Red dots represent significant proteins with increased fold-change in atelectasis compared to aerated lung; and blue for significant proteins decreased in atelectasis. (**c**, **d**) Categorical breakdown of processes in conditions of LPS(−) (**c**) or LPS(+) (**d**). *LPS* lipopolysaccharide.

Atelectasis by itself *versus* normally-aerated lung led to 130 differentially abundant (DA) proteins (up: 4, down: 126, *P* < 0.05; Fig. 1a, Fig. S2a and Table S3). The glycolytic enzyme phosphoglycerate mutase 1 (PGAM1) had the largest increased fold-change (Fig. 1a and Table 1). Systemic LPS exposure produced 174 proteins with differential abundance (up: 108, down: 66; Fig. 1b, Fig. S2b and Table S4). The proteins with largest increased fold-changes related to metabolic function: PGAM1, glyceraldehyde 3-phosphate dehydrogenase (GAPDH), lactate dehydrogenase B (LDHB), and hexokinase 2 (HK2); and to inflammation Bruton's tyrosine kinase (BTK) (Fig. 1b and Table 1). Glycoproteins THBS1, THBS2, and FSTL3, were significantly decreased irrespective of LPS (Fig. 1, Fig. S3 and Table 1).

**Table 1:** Proteins with largest and smallest fold changes in atelectatic lung.

Protein name	Entrez gene symbol	log2 fold change	*p*
**Lipopolysaccharide(−)**			
Upregulated/increased in atelectasis			
6-Phosphogluconate dehydrogenase, decarboxylating	PGD	2.367	0.105
Phosphoglycerate mutase 1	PGAM1	1.722	0.029
Importin subunit beta-1	KPNB1	1.650	0.045
Tyrosine-protein kinase CSK	CSK	1.352	0.079
Heat shock protein HSP 90-alpha/beta	HSP90AA/B1	1.252	0.187
Downregulated/decreased in atelectasis			
Thrombospondin-1	THBS1	− 1.960	< 0.001
Thrombospondin-2	THBS2	− 1.659	< 0.001
Follistatin-related protein 3	FSTL3	− 1.619	0.001
Fibrinogen	FGA FGB FGG	− 1.157	< 0.001
Histone H2A type 3	HIST3H2A	− 1.090	< 0.003
**Lipopolysaccharide(+)**			
Upregulated/increased in atelectasis			
Phosphoglycerate mutase 1	PGAM1	0.488	0.044
Glyceraldehyde-3-phosphate dehydrogenase	GAPDH	0.320	0.098
Lactate dehydrogenase B	LDHB	0.254	0.034
Bruton tyrosine kinase	BTK	0.206	0.013
C–X–C motif chemokine 11	CXCL11	0.183	0.002
Downregulated/decreased in atelectasis			
Thrombospondin-2	THBS2	− 0.343	< 0.001
Follistatin-related protein 3	FSTL3	− 0.247	< 0.001
Coiled-coil domain containing 80	CCDC80	− 0.241	0.002
Gro-beta/gamma	CXCL3 CXCL2	− 0.240	0.032
Histone H1.2	HIST1H1C	− 0.205	0.006

Functional analysis indicated 104 biological processes significantly enriched in LPS-unexposed (Table S5) and 110 in LPS-exposed atelectasis (Table S6). Atelectasis by itself yielded processes with a predominance of *negative* enrichment (58% of significant processes) related to extracellular matrix (24%), immune response (12%), tissue development (10%), stress (10%), and metabolism (26%) (Fig. 1c). In contrast, systemic LPS produced 82% of *positively* enriched significant processes in atelectatic versus aerated lung, with major processes related to immunity (36%) (Fig. 1d).

### Proteomics of atelectatic lung tissue is consistent with dysregulated immune response

**Figure 2:**
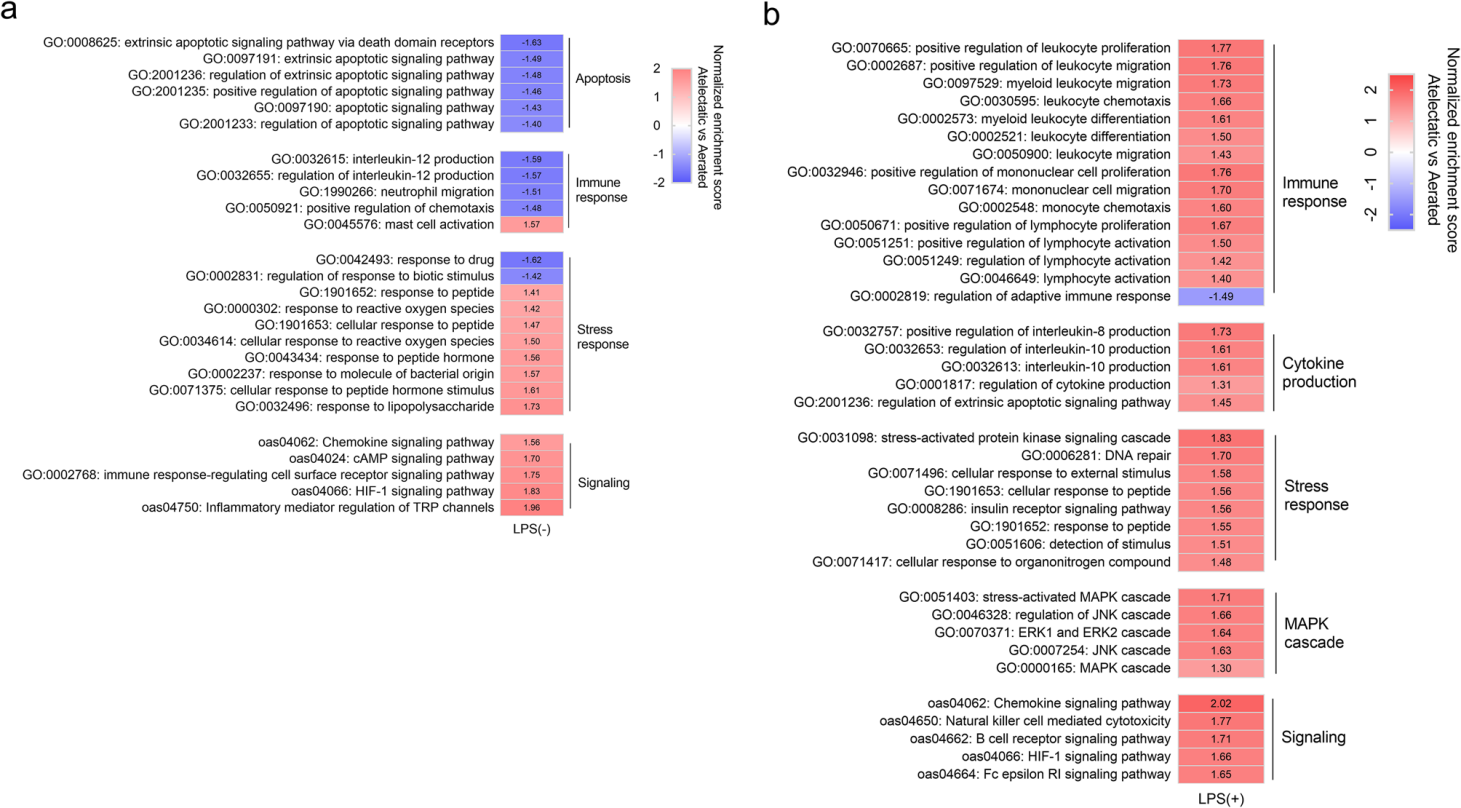
Inflammatory processes and pathways in atelectatic relative to aerated lung. (**a**) Functional analysis showing processes in apoptosis and immune response negatively enriched in atelectasis in LPS(−) conditions. Stress response and signaling of cAMP and HIF-1 present positive enrichment. (**b**) Processes and pathways related to immunity, cytokine production, stress response and MAPK cascade were positively enriched in atelectasis in LPS(+) condition. Red represents a positive normalized enrichment score. Blue represents a negative normalized enrichment score. *LPS* lipopolysaccharide, *cAMP* cyclic adenosine monophosphate, *HIF* hypoxia-inducible factors, *MAPK* mitogen-activated protein kinase.

LPS-unexposed atelectasis was associated with reduced immune response (Fig. 2a). Indeed, interleukins IL-5, IL-19 and interferon regulatory factor 1 (IRF1) were significantly decreased in the atelectatic than normally-aerated lung (Fig. 1a). Consistent with this, functional analysis revealed several negatively enriched processes in atelectasis (Fig. 2a): (a) cytokine production, *e.g.*, interleukin-12, protective against respiratory infection; (b) migration of neutrophils, the first immune cells recruited to injury sites; and (c) apoptosis-related processes.

Functional analysis also revealed the activation of stress response in atelectasis, with positively enriched response to reactive oxygen species (Fig. 2a). KEGG pathway analysis showed the involvement of signaling pathways such as HIF-1, cAMP, and chemokine, in pure atelectasis-induced immune dysfunction (Fig. 2a and Table S7).

### Systemic endotoxin induced inflammatory response in atelectatic lung

LPS exposure produced a different inflammatory proteomics phenotype in the atelectatic as compared to the normally-aerated lung. This included increased proteins for pro-inflammatory interleukins (e.g., IL-6, IL-20) and signaling molecules (e.g*.*, STAT1, MAPK12, SRC kinases FYN and LYN) (Fig. 1b). Inflammatory mediators (e.g., MPO, BTK, LYN and RAGE) and chemokines (e.g., CXCL11, CCL5, CCL8, CCL14, CCL21 and CXCL12) were also found in atelectasis with higher abundance than normally-aerated lung (Fig. 1b). Validations from ELISA measurements showed significantly increased protein levels for RAGE (Fig. S4a, *P* = 0.022) and the numerically increased CCL5 (Fig. S4b, *P* = 0.087) in LPS-exposed atelectasis, both consistent with the aptamer-based proteomics analysis.

Functional analysis confirmed the enhanced inflammatory response in atelectasis during endotoxemia (Fig. 2b). Processes related to cytokine production of interleukin-8 and interleukin-10 as well as leukocyte function, such as leukocyte migration, leukocyte differentiation and lymphocyte activation, were positively enriched in atelectatic lung. Inflammation-related signaling, including MAPK, JNK, ERK1 and ERK2 cascades (Fig. 2b), and cellular metabolism (Table S6) were also positively enriched in LPS-exposed atelectasis. KEGG pathway analysis confirmed the involvement of immune-related pathways: chemokine, B-cell receptor, Fc epsilon RI signaling, killer cell mediated toxicity (Fig. 2b); and metabolic pathways (glycolysis/gluconeogenesis) (Table S8).

### Atelectasis was related to alveolar-capillary barrier dysfunction independent of systemic endotoxin

Irrespective of systemic LPS exposure, atelectasis produced significant decrease in extracellular matrix glycoproteins (e.g., THBS1, FSTL3, THBS2), vascular endothelial growth factor (VEGFA) and fibrinogen (Fig. 1a,b and Fig. S3). LPS exposure also decreased abundance of proteins related to alveolar-capillary structures in the atelectatic versus aerated lung including FN1, NID1, PFDN5 and CDH2 (Fig. 1b). ELISA validations showed that the protein trends of THBS1 (Fig. S4c,d) and VEGFA (Fig. S4e,f) response to atelectasis were consistent with protein assessments from the aptamer-based SOMAscan method. A significant correlation was present between ELISA measurements and the aptamer-based SOMAscan proteomics measurements (Fig. S4g).

**Figure 3:**
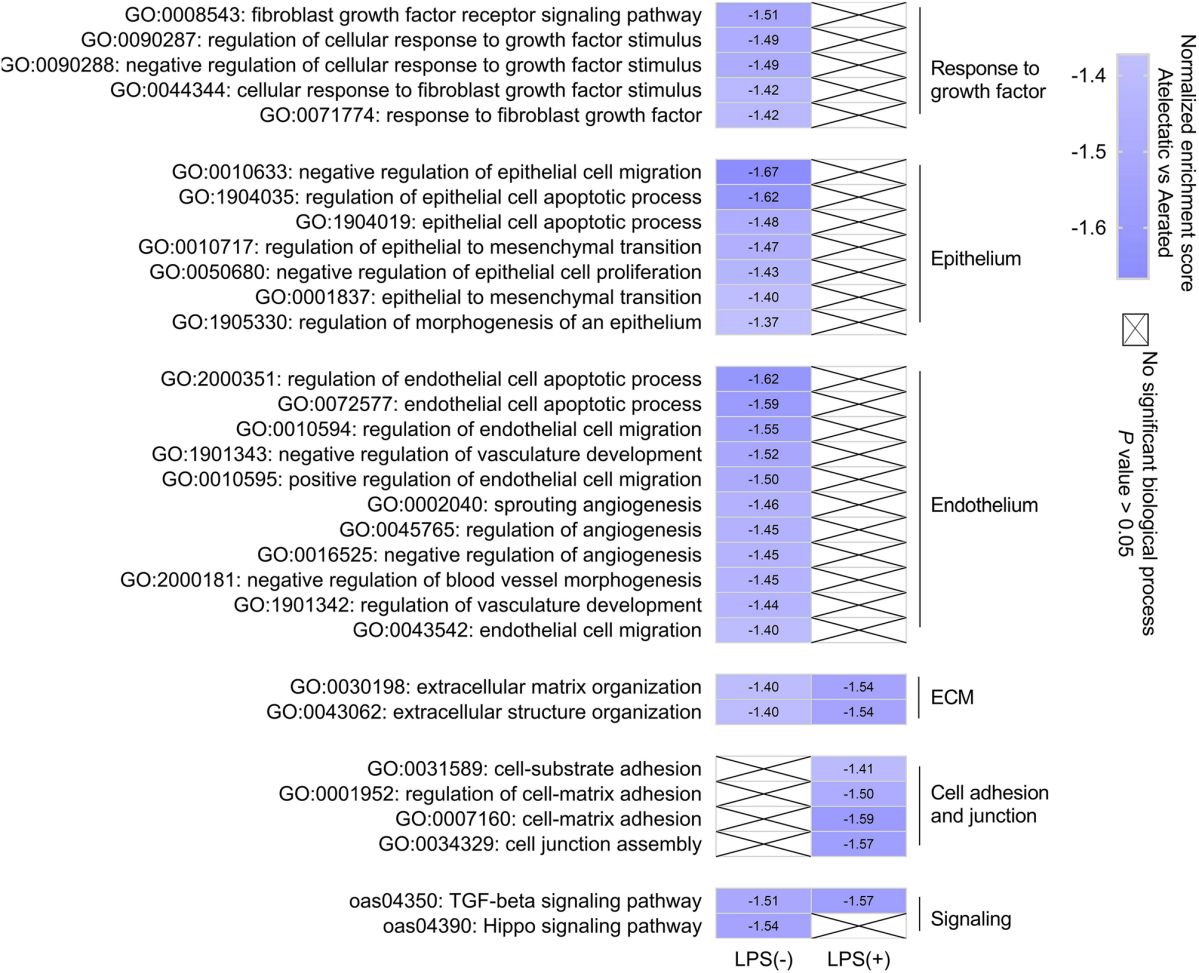
Processes and pathways related to alveolar-capillary barrier function in atelectasis. Functional analysis of proteomics data showed the negative enrichment for processes or pathways related to alveolar-capillary barrier function in LPS(−) and LPS(+) conditions. Without LPS, atelectasis was associated with negatively enriched epithelial and endothelial processes, as well as Hippo signaling pathway. With LPS, the negatively enriched processes in atelectasis were involved in cell adhesion and junction. ECM organization and TGF-β signaling were negatively enriched in LPS-exposed and unexposed atelectasis. *LPS* lipopolysaccharide, *ECM* extracellular matrix, *TGF* transforming growth factor.

Consistent with these findings, functional analysis revealed negative enrichment for organization of extracellular matrix, a network of extracellular components providing structural and biochemical support, in atelectasis regardless of LPS exposure (Fig. 3). Of note, atelectasis by itself was predominantly associated with negative enrichment for processes (including migration, proliferation and apoptosis) related to the epithelium and endothelium, major components of the alveolar-capillary barrier, and fibroblast growth factor (FGF) signaling (Fig. 3). In contrast, LPS-exposed atelectasis presented negatively enriched cell–matrix adhesion and cell junction assembly (Fig. 3).

KEGG pathway analysis revealed negative enrichment for TGF-β signaling in atelectasis irrespective of LPS exposure (Fig. 3). Hippo signaling pathway, important for lung development and regeneration, was also negatively enriched in atelectatic lung (Fig. 3). Proteins related to Hippo signaling, such as YWHAZ, TGFB2, BMP7, and FGF1, were significantly less enriched in atelectatic than aerated lung (Fig. 1a and Fig. S3). Also with lower levels in atelectasis were nuclear content for Hippo pathway effector Yes-associated protein (YAP) by immunofluorescent staining and gene expressions for YAP-responsive gene THBS1 and YAP-regulated cytoskeleton organization-associated genes ACTN1, FLNA, FLNC, SRF and RHOD by PCR (Fig. S5).

The rate constant of imaging tracer (FDG) influx from blood into lung tissue, measured by the FDG-kinetics imaging parameter K_1_, was larger in atelectasis than in aerated lung tissue, and increased more in atelectatic than aerated lung with LPS exposure (Fig. S6). Lung edema, assessed by wet/dry weight ratios, was significantly increased by LPS (Fig. S6), with no difference between atelectasis and aerated lung.

### Intersection of regional pulmonary proteomics and transcriptomics

**Figure 4:**
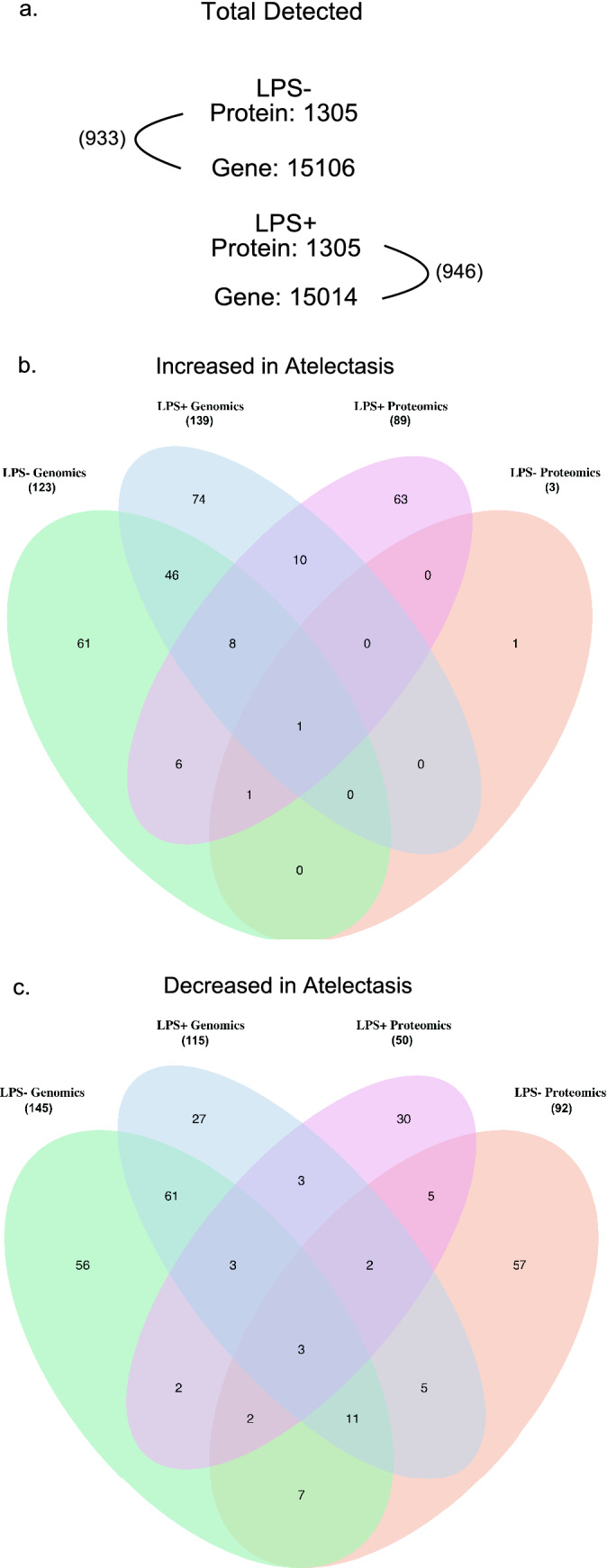
Overlap of genes and proteins in transcriptomics and proteomics. (**a**) The numbers of total detected proteins and genes in LPS(−) or LPS(+) conditions and the numbers in parentheses indicating the possible overlap between proteomics and transcriptomics. (**b**, **c**) Venn diagram showing the overlap of significantly *increased* (**b**) or *decreased* (**c**) gene/protein pairs in atelectatic relative to aerated lung in both transcriptomics and proteomics. In parenthesis, the total significant genes or proteins detected based on directionality of magnitude (increased or decreased) are displayed. *LPS* lipopolysaccharide.

There was less overlap than expected between transcriptomics and proteomics in the absence of LPS, and more overlap with LPS (*P* = 0.33 without LPS and 0.013 with LPS) (Fig. 4, Table S9 and Table S10). Inflammatory markers MPO and BTK as well as chemokines CCL5, CCL21 and CXCL12 were increased in the atelectatic versus aerated lung during LPS exposure, in both proteomics and gene expression datasets (Fig. 1b and Table S10). Hippo proteins FGF1 and YWHAZ, and glycoproteins THBS1 and FSTL3, with lower concentrations in atelectasis from proteomics datasets, had corresponding less gene expressions based on genomics analysis (Table S9 and Table S10). Biological processes related to TGF-β signaling and immune regulation, in genomics and proteomics, were negatively enriched in the atelectatic relative to aerated lung without LPS (Table S11). With LPS, cell junction assembly was negatively enriched and processes related to leukocyte migration and activation were positively enriched in atelectatic versus aerated lung (Table S11).

## Discussion

In a large animal model of single-lung atelectasis, regional lung tissue proteomics and transcriptomics analyses revealed: (1) substantial differences in protein abundance patterns occurred in the early stages of lung atelectasis with and without systemic LPS exposure; (2) decreased inflammatory proteomic signatures in atelectasis in the absence of systemic LPS; (3) increased inflammatory signatures with high levels of acute lung injury marker RAGE, chemokine CCL5 and signaling factors BTK and STAT1 during LPS exposure; (4) alveolar-capillary barrier dysfunction in atelectasis with TGF-β, Hippo signaling and extracellular matrix components such as THBS1 as potential targets; and (5) an overlapped subset of gene expression and protein abundance in atelectatic lung regions.

Evidence is limited and controversial on the biological effects of atelectasis^5,8–10^. We have recently indicated that gene expression is remarkably distinct in atelectatic versus aerated lung either in the absence or presence of systemic LPS exposure^16^. Yet, it is unknown whether and which specific components of that transcriptomics response correlate or result in ultimate protein level changes during the early stages of atelectasis.

Our one-lung ventilation model allowed for direct visual and computed tomography documentation of atelectasis, and produced expected physiological impairment of pulmonary mechanics and gas exchange, supporting the reliability of tissue samples. Resulting proteomics were consistent with a hypoxemic atelectatic lung, as expressed by the increased glycolytic enzyme PGAM1 and positively enriched HIF-1 pathway, related to hypoxic stress and relevant for epithelial, endothelial, and immune/myeloid cell response in ALI/ARDS^19^. Endotoxemia promoted further metabolic changes in atelectasis with increased glycolytic enzymes, carbon metabolism and glycolysis pathways. Previous work has validated the used aptamer-based proteomics methodology^20,21^. Such observations support the reliability of our atelectasis model and proteomics measurements.

The proteomics response was remarkably distinct in atelectatic versus aerated regions from the same lung both with and without LPS. Such differential response was relevant in two key components of lung injury: inflammation and alveolar-capillary barrier function. Without LPS, atelectasis by itself was associated with *decreased* immune function, i.e., neutrophil migration. This finding is consistent with reduced leukocyte signaling and inflammation in the dependent lung of premature lambs^18^. Of note, no significant difference in blood volume per tissue was found between atelectasis and aerated lung regions, indicating comparable blood cell numbers in vessels per tissue in both lung regions. This finding implies that the observed decreased immune function in atelectasis is likely associated with other factors rather than immune cell numbers. Indeed, our proteomics data helped explain and suggested the downregulated cellular activities, as the production of interleukin-12, with important role in neutrophil migration, was negative enriched in atelectasis.

LPS produced a switch of that proteomic inflammatory response in atelectasis from repressed towards increased. Inflammatory factors, such as IL-6, IL-10, and RAGE, all established biomarkers of ARDS^22,23^, and MPO, a neutrophil marker^24^, were significantly increased in atelectasis with LPS. Such findings are consistent with previous in vivo inflammatory assessments of regional lung inflammation^25,26^ and support the plausibility of our model and findings. The pro-inflammatory proteomics response in atelectasis was characterized by positively enriched leukocyte-associated processes including leukocyte migration. Interestingly, blood volume normalized by tissue was found lower in atelectasis than in aerated lung during LPS exposure potentially due to the effects of gravity and inhibited hypoxic pulmonary vasoconstriction. Such finding implies that our observation of enhanced inflammatory activity in atelectasis regions with LPS exposure cannot be merely assigned to a change in regional blood volume, as lower not higher inflammatory response would be expected. Indeed, our proteomics findings indicated the increased cellular responses even in the setting of reduced blood volume per tissue. It can be understood by the positively enriched interleukin-8 production and chemokines signaling, and is consistent with previous genomics data with higher levels of chemokines (e.g., CXCL10 and CCL5) in atelectasis with LPS^16^. Our findings are also consistent with increased inflammatory response of isolated atelectatic versus expanded lungs in patients undergoing esophagectomy^5^, a condition related to systemic inflammation^27^. It may be additionally important given the correlation between increased inflammatory cytokine TNF-α in atelectatic lung and worsened pulmonary outcomes after lung resection^6^.

Additionally, our proteomics data suggest potential mechanistical and treatment protein targets for such atelectasis-induced inflammatory response. These included: (a) BTK, involved with neutrophil and macrophage response in infection-induced ARDS such as influenza^28^, SARS-CoV-2^29^ and LPS/immunocomplex^30^, promotes a pro-inflammatory neutrophilic response through TLR4 signaling^30^; and (b) STAT1, an interferon-induced stimulator with function in CCL5 and CXCL11 regulation^31^. STAT1 may not only have contributed to the observed increases in CCL5 and CXCL11, but could also explain the transcriptomic findings of up-regulated interferon-stimulated genes in LPS-exposed atelectasis^16^. Overall, our results demonstrate inflammatory and corresponding mechanistic targets in atelectatic relative to aerated lung when acutely (8 h) exposed to endotoxemia.

We also found proteomic evidence for alveolar-barrier structure dysregulation in atelectasis. Of note, without LPS such response was associated to *cellular function impairment* in epithelium, endothelium and fibroblast growth signaling described in influenza and SARS-CoV-2-induced ARDS^32^. In contrast, dysfunction in *cell junction assembly* and *cell–matrix/substrate adhesion*, processes found in lung injury^33,34^, predominated in atelectasis with LPS. Negatively enriched TGF-β signaling, important in alveolar type-2 cell regeneration during LPS-induced murine lung injury^35^, further suggested barrier dysfunction in atelectasis. Our results are consistent with previous observations in the dependent lung of preterm lambs and recent transcriptomics findings in atelectasis^16,18^. The rate of imaging tracer influx from blood into lung tissues (K_1_) larger in atelectasis versus aerated lung regions could partially reflected ultrastructural or functional changes occurring in the alveolar-capillary barrier in atelectasis. This possibility is also consistent with the larger proportional increase of K_1_ in atelectatic than aerated tissue with LPS exposure. In line with such findings, RAGE, an injury marker of type-1 cell in acute lung injury^36^, was significantly increased in atelectasis during LPS exposure. Overall, given that our tissue samples derived from a non-ventilated lung, our findings imply that atelectasis, either with or without LPS exposure, could contribute to alveolar-capillary barrier dysfunction even in the absence of biomechanical injurious forces.

Of note, the Hippo pathway was significantly enriched in atelectatic versus aerated lungs. This pathway is associated with epithelial proliferation after lung injury^37^, and consequently a potential factor for alveolar-capillary barrier dysfunction. As an important effector of Hippo pathway, Yes-associated protein (YAP) was lower in atelectasis, validated by immunofluorescent staining and supported by the lower expression of YAP-responsive gene THBS1. Recently, YAP has been reported as involved in barrier function via regulation of cytoskeleton dynamics^38^. Our PCR validations confirmed the lower expression for YAP regulated cytoskeleton organization-associated genes (*e.g.*, ACTN1, FLNA, FLNC, SRF and RHOD). Together these data reveals the consistency of proteomics with recent transcriptomics in barrier dysfunction, and provides further support to the link of Hippo-YAP signaling with atelectasis^16^. Such findings suggest the role of the Hippo pathway not only as a possible factor in the mechanism of atelectasis-related lung injury, but also as a potential therapeutic target aiming at barrier function improvement.

Additionally, extracellular matrix glycoproteins (e.g., THBS1 overlapped in transcriptomics and proteomics as well as validated in gene and protein levels) were lower in atelectatic than aerated lung with and without LPS. THBS1, associated with ventilator-induced lung injury^39^, and THBS2, with function in extracellular matrix assembly^40^, are both related to lung remodeling^41^. FSTL3, involved with epithelial regulation, is lower in patients with asthma or bronchoconstriction than in healthy individuals^42^. VEGFA, a major glycoprotein inducer of integrins and downstream angiogenesis processes, has been involved in the pathogenesis of ARDS during oesophagectomy^43^ and decreased in acute models of LPS-induced lung injury and early ARDS^44,45^. These findings support the ability of our model to reproduce previous lung injury observations, and present novel findings suggesting the presence of dysregulated barrier function by atelectasis regardless of LPS exposure.

Of note, there has been substantial controversy on the use of ventilatory management strategies directed to minimization of atelectasis both in surgical and critically-ill patients^1,4^. Biological information has been scant, with experimental studies reflecting controversial results in humans^46,47^, and the debate centered on results of specific clinical trials and global physiological studies. Using large animal models mimicking clinical settings and representing well the human condition structurally and physiologically, our demonstration of substantial transcriptomics and proteomics differences in atelectasis provides biological plausibility to optimal recruitment techniques beyond their specific effect on biomechanical forces.

There are limitations in our study. The sample size limits power to detect significant differences in regional protein concentrations. Our large animal model focused on the early stages of atelectasis with timeframe of 8 h, which is relevant to the perioperative period involving surgeries using one-lung ventilation such as lung resection, pneumonectomy, and thoracoabdominal aortic aneurysm repair. Thus, results are expected to relate to mechanisms of lung injury in the early stages of atelectasis and did not address the long-term effects of atelectasis. Our analysis does not identify the specific cells contributing to the proteomics findings. While functional analysis suggested cell subtypes, further studies will be required to determine cell-specific responses. Our large animal model represents clinical conditions of one-lung ventilation with mild-moderate histological lung injury^16^. Accordingly, endothelial and epithelial cell death following atelectasis has not been detected at this early time point. Further investigation extending phenotypic characterization and molecular biology is necessary to gain full understanding in atelectasis-associated alveolar barrier dysregulation. Our findings are restricted to the utilized model and do not describe all mechanisms and insults found in patients developing lung injury.

In conclusion, atelectatic tissue presented proteomics patterns consistent with a dysregulated immune response. Additional systemic endotoxin shifted this into an enhanced regional inflammatory response, *e.g.*, increased acute lung injury marker RAGE, chemokine CCL5 and signaling factors BTK and STAT1 in atelectatic versus aerated lung. Alveolar-capillary barrier dysfunction in atelectatic lung regions was independent of LPS exposure. TGF-β signaling, Hippo pathway and extracellular matrix components (*e.g.*, THBS1) can be potential culprits for the dysregulated barrier function in atelectasis.

## Methods

Experimental animal protocols were approved by the Massachusetts General Hospital's Subcommittee on Research Animal Care and the Institutional Animal Care and Use Committee (IACUC number: 2006N000129, Boston, Massachusetts) and in accordance with the "Guide for the Care and Use of Laboratory Animals" published by the National Institutes of Health (publ. no. 86-23, revised 1996)^48^ and in adherence to ARRIVE guidelines^49^. Methods are fully described in the supplement and experimental approaches are summarized in Fig. S1.

### Experimental setup

Twelve female sheep (18.2 ± 2.3 kg) underwent general anesthesia, intubation, mechanical ventilation with one-lung ventilation. Left lung atelectasis was created using a left bronchial blocker and left lateral thoracotomy to allow for passive lung collapse. The right lung was ventilated with tidal volume (V_T_) = 10 ml/kg, positive end-expiratory pressure (PEEP) = 2 cm H_2_O and inspiratory-to-expiratory time ratio of 1:2. The fraction of inhaled O_2_ (F_i_O_2_) was initially set at 0.3 and increased to maintain oxygen saturation > 88%. Once one-lung ventilation was established, animals were turned laterally and ventilated for 8 h. Sheep were divided into LPS-unexposed (LPS-, n = 6) and LPS-exposed (LPS+, n = 6) groups. LPS exposure consisted of an intravenous infusion at 10 ng/kg/min (*Escherichia coli* O55:B5, List Biologic Laboratories Inc., Campbell, CA) for 30 min followed by 5 ng/kg/min for the remainder of the experiment.

### Image-guided tissue samples

At the end of the 8-h experiment, animals were euthanized under deep anesthesia. Lung tissue was harvested from the atelectatic and normally-aerated regions using computed tomography (CT) image-guided sampling. The CT scan confirmed left lung samples with collapse status and the right upper lung samples with normal aeration. Positron emission tomography (PET) was used to estimate fractional blood volume and the rate constant of imaging tracer 18F-fluorodeoxyglucose (FDG) influx from blood into lung tissue (K_1_) in both atelectatic and aerated lung regions. All lung samples were immediately stored at − 80 °C for further proteomics analysis.

### SOMAscan proteomics assay and validation

Lung tissue samples from atelectatic and aerated regions were collected for SOMAscan, a multiplex aptamer-based proteomics assay (Somalogic, Boulder, CO). SOMAscan was run on the samples to capture 1305 protein analytes, using SOMAmer reagents^50^. The samples were prepared following recommended sampling and handling procedure for both tissue types^50,51^. They were then run in the Somalogic certified assay site at the BIDMC genomics, proteomics, bioinformatics and systems biology center at Beth Israel Deaconess medical center along with pooled and quality control samples according to the manufacturer's well-established protocols. The limma package in R (version 3.5.2)^52^, a mixed effects linear model, was used to run on the normalized SOMAscan output data in Relative Fluorescence Units. Validations of findings from the aptamer-based proteomics assay, including protein levels, gene expressions and lung edema were performed in atelectatic and aerated lung tissues.

### Functional analysis

All proteins from the tissue limma model output were ranked based on a weight comprised of log fold change * (1-p value). Gene ontology (GO) and Kyoto Encyclopedia of Genes and Genomics (KEGG)^53^ were used to perform Gene Set Enrichment analysis (GSEA), using a ranked list of all detected proteins^54^. Gene Set Enrichment analysis gave output ontologies and pathways with leading edge analysis statistics. All ontology types were considered: molecular function (MF), cellular component (CC) and biological process (BP). The calculated enrichment score represents the degree to which a set is overrepresented at the top or bottom of a ranked list of proteins using the Kolmogorov–Smirnov summation Statistic. A positive enrichment score indicates increased enrichment in the atelectatic relative to aerated lung. The Kolmogorov–Smirnov test is a non-parametric test against the null distribution. The gene ontology database was used to bin proteins that co-enriched annotated ontologies to understand functional characteristics. The KEGG database includes named pathways with known implication in disease. The process and pathway analysis used in-house modified functional analysis scripts from BcBiornaseq and Deseq2 packages in R statistical software^55,56^. Overlapping lists were generated in R by comparing the proteomics results with 2363 differentially expressed genes without LPS, 3767 genes with LPS, and corresponding processes.

### Statistical analysis

Data are presented as mean ± SD if normally distributed and median and interquartile interval (25 to 75%) otherwise. The limma package in R (version 3.5.2), a mixed effects linear model, was run on the raw SOMAscan output data in Relative Fluorescence Units. In this hypothesis-generating, exploratory analysis^57^, we explored the effect of atelectatic compared to aerated lung using *p* < 0.05. The limma model analysis output is reported in the supplement containing the log_2_foldchange, calculated beta, *p*-value, and adjusted *p*-value. A paired, two-tailed Student's t-test was used for comparison between atelectasis and aerated lung in GraphPad Prism software v.7.0 (GraphPad Software, USA). Correlation analysis was evaluated by Pearson’s correlation analysis. *P* < 0.05 were considered statistically significant.

## Supplementary Information

Supplementary Information.

Supplementary Tables.

## Data Availability

Complete datasets for the linear model output, processes and pathway analysis, genomics results, and validation are included in the supplement.
