# Supplementary material for: Proteomics of lung tissue reveals differences in inflammation and alveolar-capillary barrier response between atelectasis and aerated regions
Source: Sci Rep. 2022 Apr 29;12:7065. doi: 10.1038/s41598-022-11045-7 (PMC9053128; doi:10.1038/s41598-022-11045-7)
Supplement: Supplementary file 1 — Supplementary Information. [file 41598_2022_11045_MOESM1_ESM.docx]

**Proteomics of Lung Tissue Reveals Differences in Inflammation and Alveolar-Capillary Barrier Response between Atelectasis and Aerated Regions**

Azman Rashid, MSc^1,2^, Congli Zeng, MD, PhD^1,3^*, Gabriel Motta-Ribeiro, ScD^1,4^, Simon T. Dillon, PhD^5^, Towia A. Libermann, PhD^5^, Marcos Adriano Lessa, MD, PhD^1^, Aranya Bagchi, MD^1^, John Hutchinson, PhD^6^, Marcos F. Vidal Melo, MD, PhD^1,3^

**Supplementary Digital Content**

**Materials and Methods**

Animal studies were performed under Massachusetts General Hospital’s Subcommittee on Research Animal Care (IACUC number: 2006N000129) and according to "Guide for the Care and Use of Laboratory Animals" published by the National Institutes of Health (publ. no. 86-23, revised 1996)^1^. Experimental study design and procedure was conducted and reported in adherence to ARRIVE guidelines^2^.

**Animal model**

Twelve female sheep (18.2 ± 2.3 kg) underwent general anesthesia, intubation and mechanical ventilation in right lateral position. General anesthesia was performed with infusion of propofol (5mg/kg) and fentanyl (10 ug/kg). Paralysis was maintained with a bolus and continuous injection of vecuronium (0.5 mg/kg). Left lung atelectasis was created using a left bronchial blocker and a left lateral thoracotomy to allow for passive left lung collapse. One-lung ventilation of the right lung used the following settings: tidal volume (V_T_) of 10 ml/kg, positive end-expiratory pressure (PEEP) of 2 cm H_2_O and inspiratory-to-expiratory ratio was 1:2. The fraction of inhaled O_2_ (F_i_O_2_) was 0.3 and increased to maintain oxygen saturation greater than 88%. PEEP was increased in increments of one cm H_2_O if normal blood gasses were not maintained at an F_i_O_2_ of 1. The initial respiratory rate was twenty-five breaths/min and titrated to maintain normocapnia (arterial carbon partial pressure between 32 and 45 mmHg). Once one lung ventilation was established, animals were ventilated for eight hours.

Twelve sheep were divided into two groups: Lipopolysaccharide endotoxin exposed (LPS+) and LPS unexposed (LPS-) group. An intravenous infusion of endotoxin (10ng/kg/min, Escherichia coli O55:B5, List Biologic Laboratories Inc., Campbell, CA) was administered to six of the sheep in the LPS+ group (18.3 ± 2.4 kg) in the first thirty minutes. Continuous infusion was administrated for the rest of the experiment (5 ng/kg). Reduction of LPS infusion and lactated Ringer’s solution was used to manage proper cardiac function and hemodynamics with the systemic inflammatory stimulus. The other six sheep were in the LPS- group (18.1 ± 2.4 kg). After stabilization, physiological parameters, arterial and central venous blood samples were acquired.

**Image-Guided Tissue Samples**

At the end of the 8-hour experiment, animals were euthanized under deep anesthesia. Lung tissue was harvested from atelectatic and aerated regions using Computed tomography (CT) image-guided sampling for proteomics analysis. The CT scan confirmed left lung samples with collapse status and the right upper lung samples appeared normally aerated. Positron emission tomography (PET) was used to estimate fractional blood volume and the rate constant of imaging tracer 18F-fluorodeoxyglucose (FDG) influx from blood into lung tissue (K_1_) in both atelectatic and aerated lung regions-of-interest. Details of methods for the PET imaging in fractional blood volume and infiltration rate, as well as CT scan acquisition and computational analysis of strain and aeration (F_gas_) have been described previously^3–5^.

**SOMAscan Proteomics Assay**

Lung tissue samples were collected from twelve sheep that underwent one-lung ventilation. SOMAscan (Somalogic, Boulder, CO), a multiplex aptamer-based assay, was run on the samples to capture 1305 protein analytes, using SOMAmer reagents^6^. The samples were prepared following recommended sampling and handling procedure for both tissue types^6,7^. They were then run in the Somalogic certified assay site at the BIDMC genomics, proteomics, bioinformatics and systems biology center at Beth Israel Deaconess medical center along with pooled and quality control samples according to the manufacturer’s well-established protocols.

**Linear Model**

Using the limma package in R (version 3.5.2)^8^, a mixed effects linear model was run on the normalized SOMAscan output data in Relative Fluorescence Units (RFU). In this exploratory analysis, we explored the effect of atelectatic compared to aerated lung, accounting for paired sheep sampling (*p* < 0.05). A moderated, paired t-test was used to account for paired sheep sampling. In the equation:

$$\boldsymbol{E}\left( \boldsymbol{y}_{\boldsymbol{ij}} \right)=\beta_{0}+\beta_{1} X_{i,j}+e_{i,j}$$

# $\boldsymbol{E}\left( \boldsymbol{y}_{\boldsymbol{ij}} \right)$ represents the log_2_ of the raw Somascan measurement in RFU units; exp(β_1_) and exp(β_o_) are the calculated fold change for each detected protein i comparing atelectatic to the reference aerated lung, respectively. X_i,j_ is a binary indicator variable equal to 1 when sample i is from the atelectatic tissue, and $\mathbf{e}_{\mathbf{ij}}$ is the random effect for each sheep j following a normal distribution $\mathbf{e}_{\mathbf{i,j}}\boldsymbol{\sim N(0,}$σ^2^). The model was run on endotoxin exposed (LPS+) and non-endotoxic sheep (LPS-) separately and follows a log normal distribution: the log_2_ of the positive SOMAscan measurement can take any positive or negative value. An unadjusted alpha of 0.05 was used in the use of hypothesis-generating exploratory GSEA methods rather than a Bonferroni-adjusted alpha threshold. The limma model analysis output is reported in the supplement containing the log_2_foldchange from the calculated beta, *p*-value, and adjusted *p*-value.

**Functional Analysis**

All proteins from the tissue limma model output were ranked based on a weight comprised of log fold change * (1-p value). Gene ontology (GO) and Kyoto Encyclopedia of Genes and Genomics (KEGG) databases were used to perform Gene Set Enrichment analysis (GSEA), using a ranked list of all detected proteins^9^. Gene Set Enrichment analysis gave output ontologies and pathways with leading edge analysis statistics. All ontology types were considered: molecular function (MF), cellular component (CC) and biological process (BP). The calculated enrichment score represents the degree to which a set is overrepresented at the top or bottom of a ranked list of proteins using the Kolmogorov-Smirnov summation Statistic. A positive enrichment score indicates increased enrichment in the atelectatic relative to aerated lung. The Kolmogorov-Smirnov test is a non-parametric test against the null distribution. The gene ontology database was used to bin proteins that co-enriched annotated ontologies to understand functional characteristics. The KEGG database of biological pathways includes named pathways with known implication in disease. The process and pathway analysis used in-house modified and functional analysis scripts from BcBiornaseq and Deseq2 packages in R statistical software^10,11^.

**Comparison with RNA-seq Analysis**

Transcriptomics data in atelectatic relative to aerated lung by Illumina RNA-sequencing analysis was obtained from the previous publication^12^. The data was analyzed using Deseq2, generating differential expression and gene set enrichment analysis. R (version 3.5.2) scripts were used to generate overlapping lists comparing 2363 differentially expressed genes without LPS, 3767 genes with LPS, and corresponding processes with the proteomics results in this study. Of that subset of total assayed genes/proteins, we looked at differentially expressed genes and differentially abundant proteins. A hypergeometric test was used to compare if the overlap between genomics and proteomics was more or less than expected. Comparative lists looked at the overlap between genes and proteins in LPS- and LPS+ conditions, described in Figure 4, Table S9 and S10.

**Validation of proteomics data**

Regional lung tissues were harvested from atelectatic and normally aerated regions. A subset of proteins of interest including AGER/RAGE, CCL5/RANTES, THBS1, and VEGFA (Abcam Inc. Cambridge, United Kingdom, RayBiotech, Inc., Norcross, GA, USA, MyBioSource, San Diego, CA, USA) were measured in duplicates by using the commercially available enzyme-linked immunosorbent assay (ELISA) kits according to the manufacturer's protocol. (Figure S4). Protein levels were measured at 450 nm using a microplate reader and normalized using total protein concentration detected in tissue homogenate. The linear correlation between the SOMAscan assay and ELISA results transformed by log_10_ was calculated using Pearson's correlation analysis. Protein distribution of YAP was assessed on 5 µm paraffin sections from aerated and atelectatic regions of LPS treated and untreated lungs by using immunofluorescent staining according to the manufacturer's protocol. Gene expressions of YAP-responsive gene THBS1 and actin cytoskeleton organization related genes (including ACTN1, FLNA, FLNC, SRF and ROHD) were also measured by real-time polymerase chain reaction according to the manufacturer's protocol. Lung edema in samples from the atelectatic and aerated lungs were estimated by wet/dry weight ratios, which were calculated as the average of the ratios of three samples from each region.

**Statistical Analysis**

Data are presented as mean ± SD if normally distributed and median and interquartile interval (25 to 75%) otherwise. Using the limma package in R (version 3.5.2), a mixed effects linear model was run on the raw SOMAscan output data in Relative Fluorescence Units. In this hypothesis-generating, exploratory analysis, we explored the effect of atelectatic compared to aerated lung using *p* < 0.05. The limma model analysis output is reported in the supplement containing the log_2_foldchange, calculated beta, *p*-value, and adjusted *p*-value. Protein levels, gene expressions, wet/dry weight ratios and K_1_ between atelectasis and aerated lung were compared by a paired, two-tailed Student's t-test in GraphPad Prism software v.7.0 (GraphPad Software, USA). Correlation analysis between SOMAscan analysis and ELISA results transformed by log_10_ was evaluated by Pearson’s correlation analysis. *P* values less than 0.05 were considered statistically significant.

**References**

1. National Research Council (US) Committee for the Update of the Guide for the Care and Use of Laboratory Animals. *Guide for the Care and Use of Laboratory Animals*. (National Academies Press (US), 2011).

2. Percie du Sert, N. *et al.* The ARRIVE guidelines 2.0: Updated guidelines for reporting animal research. *PLoS Biol* **18**, e3000410 (2020).

3. Wellman, T. J. *et al.* Lung Metabolic Activation as an Early Biomarker of Acute Respiratory Distress Syndrome and Local Gene Expression Heterogeneity: *Anesthesiology* **125**, 992–1004 (2016).

4. Costa, E. L. V. *et al.* Mild Endotoxemia during Mechanical Ventilation Produces Spatially Heterogeneous Pulmonary Neutrophilic Inflammation in Sheep: *Anesthesiology* **112**, 658–669 (2010).

5. Motta-Ribeiro, G. C. *et al.* Deterioration of Regional Lung Strain and Inflammation during Early Lung Injury. *Am J Respir Crit Care Med* **198**, 891–902 (2018).

6. Kraemer, S. *et al.* From SOMAmer-based biomarker discovery to diagnostic and clinical applications: a SOMAmer-based, streamlined multiplex proteomic assay. *PLoS One* **6**, e26332 (2011).

7. Shubin, A. V. *et al.* Blood proteome profiling using aptamer-based technology for rejection biomarker discovery in transplantation. *Sci Data* **6**, 314 (2019).

8. Ritchie, M. E. *et al.* limma powers differential expression analyses for RNA-sequencing and microarray studies. *Nucleic Acids Res* **43**, e47 (2015).

9. Subramanian, A. *et al.* Gene set enrichment analysis: a knowledge-based approach for interpreting genome-wide expression profiles. *Proc Natl Acad Sci U S A* **102**, 15545–15550 (2005).

10. Steinbaugh, M. J. *et al.* bcbioRNASeq: R package for bcbio RNA-seq analysis. *F1000Res* **6**, 1976 (2018).

11. Love, M. I., Huber, W. & Anders, S. Moderated estimation of fold change and dispersion for RNA-seq data with DESeq2. *Genome Biol* **15**, 550 (2014).

12. Zeng, C. *et al.* Lung Atelectasis Promotes Immune and Barrier Dysfunction as Revealed by Transcriptome Sequencing in Female Sheep. *Anesthesiology* **133**, 1060–1076 (2020).

**Supplementary Figures**

**Figure S1** **Workflow for Experiment and Analysis.**

**Figure S2** **Principal component analysis plot for lung samples in conditions of LPS(-) (a) and LPS(+) (b).** Variables are reduced in dimensionality and clustered based on principal component variables. PC1 and PC2 refer to the two dimensions that explain the highest variability. Aerated samples are blue while atelectatic samples are shown in red. The ellipse shows the 95% confidence interval of the points; the size is directly proportional to the variance. LPS = lipopolysaccharide.

**Figure S3. Heatmaps for top ten significant proteins** in LPS(-) (a) and LPS(+) (b) conditions. THBS1, THBS2 and FSTL3 are significantly decreased in atelectasis in both LPS-exposed and unexposed conditions. The heatmaps show clustering of atelectatic and aerated samples. The ID for each raw lung sample is presented. The same number represents the same sheep from which atelectatic and aerated lung samples were taken. LPS = lipopolysaccharide; THBS = thrombospondin; FSTL3 = follistatin like 3.

**
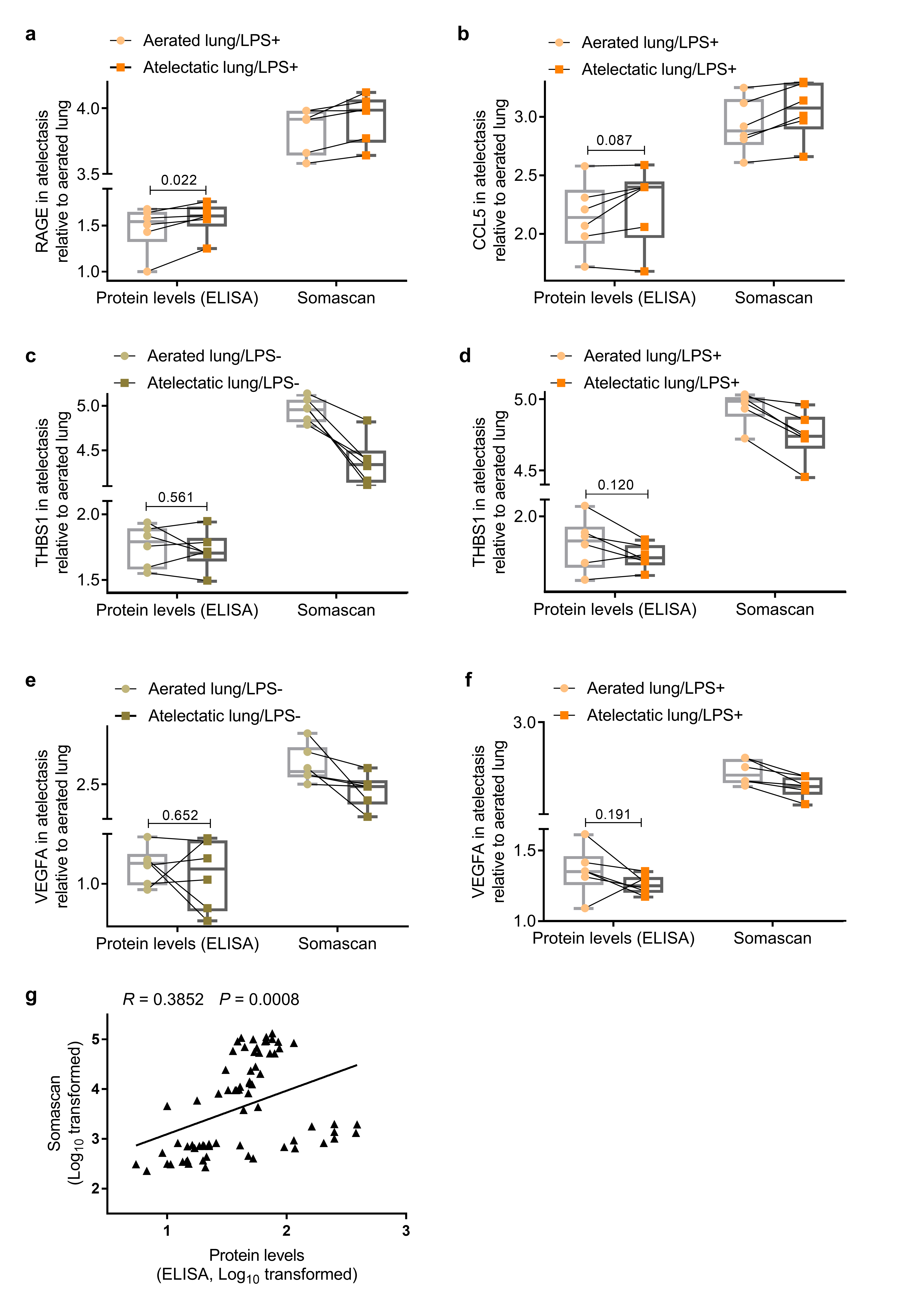
**

**Figure S4 The validations at protein levels by ELISA.** (a) The protein levels of RAGE in LPS-exposed atelectasis were significantly decreased when compared to LPS-exposed aerated lung tissues. (b-f) Although without statistical significance for other validated proteins, the changes in trends at protein levels by ELISA were consistent with those observed in Somascan proteomics analysis. (g) by Log-transformed protein levels from ELISA measurements and Somascan proteomics analysis were positively correlated. RAGE = receptor for advanced glycation endproducts; CCL5 = C-C motif chemokine ligand 5; THBS1 = thrombospondin 1; VEGFA = vascular endothelial growth factor A; LPS = lipopolysaccharide; ELISA = enzyme-linked immunosorbent assay.


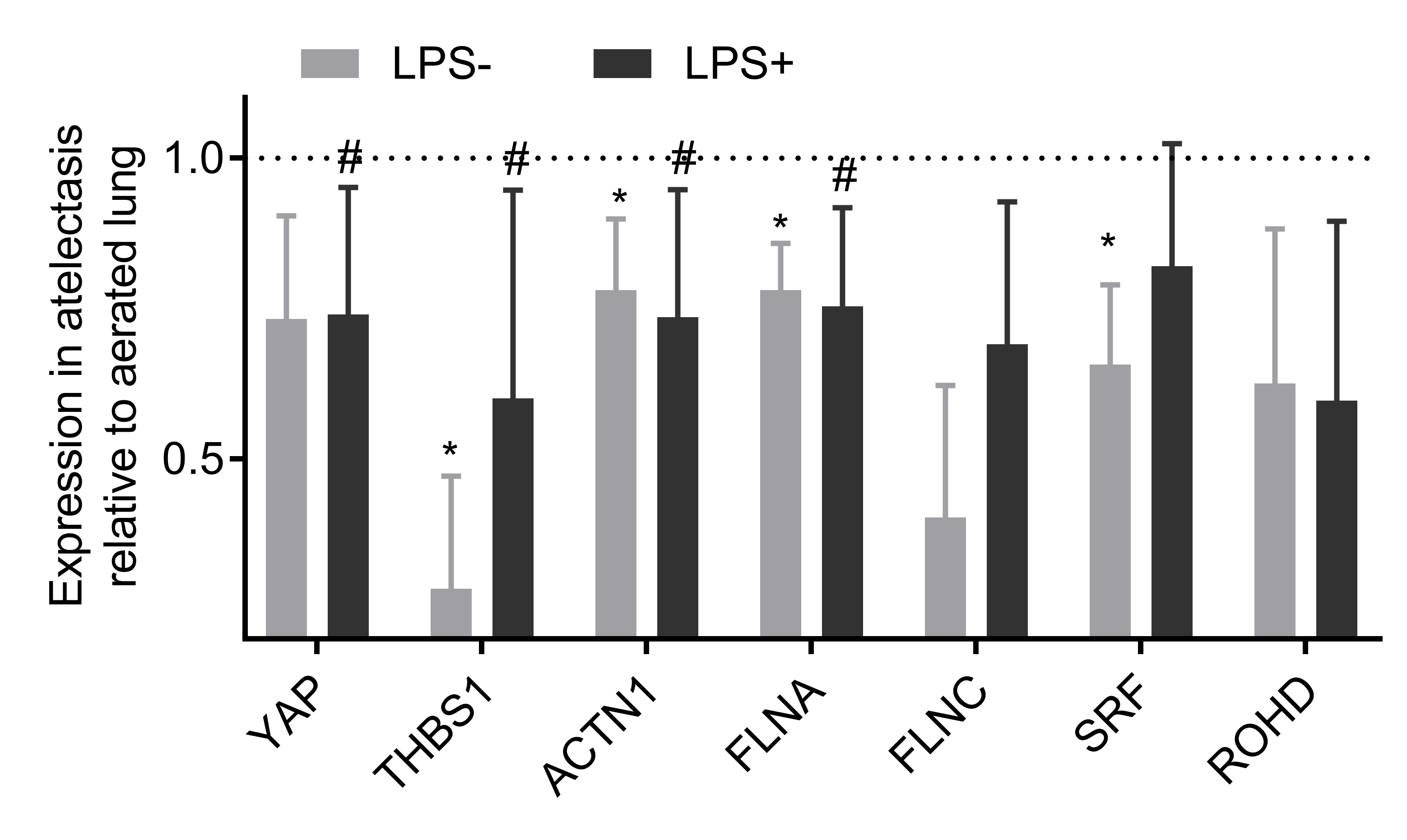


**Figure S5 The expression of factors related to barrier function.** Hippo pathway effector YAP was measured by immunofluorescent staining with less nuclear content in atelectasis than in aerated regions. PCR validations showed the decreased gene expression for YAP-responsive gene THBS1 and cytoskeleton organization-associated genes including ACTN1, FLNA, FLNC, SRF and ROHD. * *P* < 0.05; atelectasis versus aerated lung tissues without LPS (LPS-). # *P* < 0.05; atelectasis versus aerated lung tissues with LPS (LPS+). YAP = yes-associated protein; THBS1 = thrombospondin 1; ACTN1 = actinin alpha 1; FLNA = filamin A; FLNC = filamin C; SRF = serum response factor; RHOD = Ras homolog family member D; LPS = lipopolysaccharide; PCR = polymerase chain reaction.


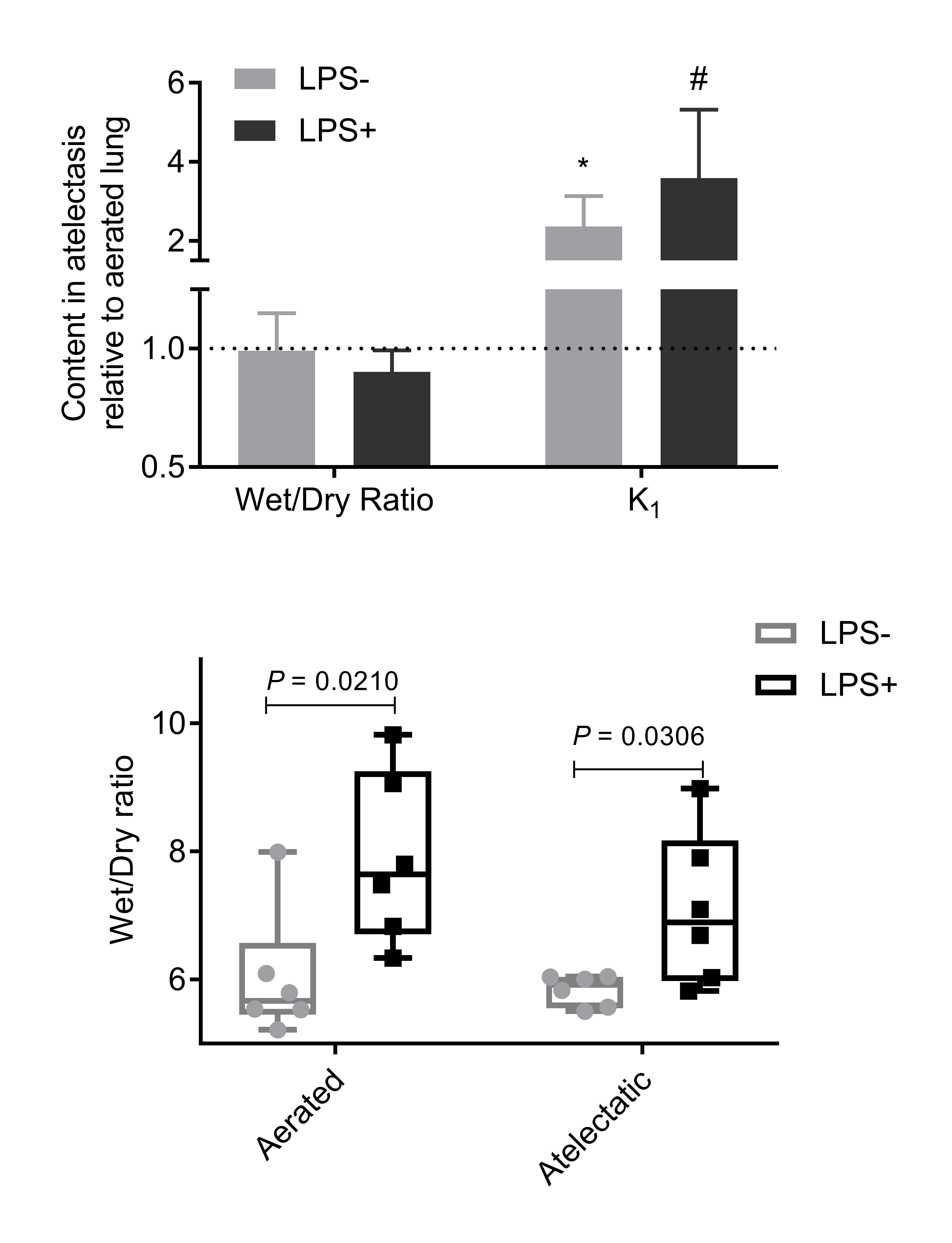


**Figure S6 The functional assessment of barrier permeability.** Lung edema, determined by wet/dry weight ratios, was significantly increased with LPS (*P* = 0.0210 in aerated and *P* = 0.0306 in atelectatic lung, detailed in lower panel figure) while no difference between atelectasis and aerated lung regions. The infiltration rate of imaging tracer from blood into lung tissue, assessed by PET-FDG imaging parameter K_1_, was dramatically increased in atelectasis irrespective of LPS exposure. * *P* < 0.05; infiltration rate into atelectasis versus into aerated lung tissues without LPS (LPS-). # *P* < 0.05; infiltration rate into atelectasis versus into aerated lung tissues with LPS (LPS+). LPS = lipopolysaccharide.

ELISA measurements for four selected proteins compared to their respective raw Somascan measurements

**Supplementary Tables**

**Table S1** Physiological variables for 8 hour one-lung ventilation. V_T_ = tidal volume, PEEP = positive end-expiratory pressure, MAP = mean airway pressure, P_aO2_/F_iO2_ = ratio of arterial oxygen partial pressure to Fractional inspired oxygen, P_vO2_ = mixed venous partial pressure of oxygen, ET_CO2_ = end tidal carbon dioxide, HR = heart rate, SBP = systolic blood pressure, DBP = diastolic blood pressure, MPAP = mean pulmonary artery pressure, CO = cardiac output.

|  | **Lipopolysaccharide(-)** | | | **Lipopolysaccharide(+)** | | |
| --- | --- | --- | --- | --- | --- | --- |
|  | **Baseline** | **Atelectasis-0h** | **Atelectasis-8h** | **Baseline** | **Atelectasis-0h** | **Atelectasis-8h** |
| VT, ml/kg | 9.7±0.3 | 9.6±0.3 | 10.0±0.7 | 9.5±0.2 | 9.4±0.1 | 9.3±0.4 |
|  |  |  |  |  |  |  |
| PEEP, cm H_2_O | 2±0 | 2±0 | 2±0 | 2±0 | 2±0 | 5±3 |
| MAP, cm H_2_O | 6.17±0.52 | 7.67±0.52 | 8.8±2.68 | 6.33±0.52 | 8.83±1.17 | 15±4.29 |
| P_aO2_/F_iO2_, mm Hg | 299±46 | 175±24 | 146±42 | 351±91 | 152±21 | 81±30 |
| P_vO2_, mm Hg | 47±5 | 44±3 | 42±5 | 49±8 | 42±5 | 43±17 |
| Bicarbonate, mM | 23.40±1.65 | 24.56±2.82 | 22.65±3.28 | 24.30±2.95 | 23.63±2.95 | 16.45±5.88 |
| ET_CO2_, mm Hg | 38±4 | 34±5 | 34±5 | 26±2 | 32±3 | 32±3 |
| HR, beats/min | 123±25 | 113±14 | 112±6 | 114±27 | 115±19 | 137±52 |
| SBP, mm Hg | 112±12 | 109±20 | 112±19 | 110±10 | 74±16 | 68±14 |
| DBP, mm Hg | 79±13 | 74±16 | 68±11 | 74±10 | 83.83±12 | 54.2±12 |
| MPAP, mm Hg | 19±3 | 22±2 | 22±3 | 16±3 | 23±2 | 39±6 |
| CO, L/min | 3.1±0.7 | 3.3±0.8 | 3.5±0.9 | 3.0±0.7 | 3.2±0.4 | 2.1±0.8 |

**Table S2** The lung strain, aeration (F_gas_, gas fraction) and blood volume for atelectatic and aerated lung.

|  | **Lipopolysaccharide(-)** | | **Lipopolysaccharide(+)** | | **Overall** | |
| --- | --- | --- | --- | --- | --- | --- |
| **Lung** | **Atelectatic** | **Aerated** | **Atelectatic** | **Aerated** | **Atelectatic** | **Aerated** |
| Strain | 0±0 | 0.59±0.44 | 0±0 | 0.43±0.20 | 0±0 | 0.51±0.34 |
| Aeration, F_gas_ | -0.03±0.01 | 0.61±0.07 | -0.02±0.01 | 0.61±0.09 | -0.02±0.01 | 0.61±0.08 |
| Blood Volume | 0.064±0.019 | 0.067±0.029 | 0.075±0.026 | 0.135±0.056 | 0.069±0.023 | 0.10±0.042 |

**Table S3** List of limma-output of detected proteins with reported log_2_foldchange, beta, *p*-value and adjusted *p*-value for Lipopolysacharide(-) conditions. (Please find in .xlsx file "Supplementary Dataset. xlsx".)

**Table S4** List of limma-output detected proteins with reported log_2_foldchange, beta, *p*-value and adjusted *p*-value for Lipopolysacharide(+) conditions. (Please find in .xlsx file "Supplementary Dataset. xlsx".)

**Table S5** Total list of detected Gene-ontology (GO) processes with reported normalized enrichment score, *p*-value and adjusted *p*-value for Lipopolysacharide(-) conditions. (Please find in .xlsx file "Supplementary Dataset. xlsx".)

**Table S6** Total list of detected Gene-ontology processes with reported normalized enrichment score, *p*-value and adjusted *p*-value for Lipopolysacharide(+) conditions. (Please find in .xlsx file "Supplementary Dataset. xlsx".)

**Table S7** Total list of detected KEGG pathways with reported normalized enrichment score, *p*-value and adjusted *p*-value for Lipopolysacharide(-) conditions. (Please find in .xlsx file "Supplementary Dataset. xlsx".)

**Table S8** Total list of detected KEGG pathways with reported normalized enrichment score, *p*-value and adjusted *p*-value for Lipopolysacharide(+) conditions. (Please find in .xlsx file "Supplementary Dataset. xlsx".)

**Table S9** Overlap of significant genes and proteins detected in Lipopolysacharide(-) conditions. ID: gene or protein name, Log_2_FC: log_2_ fold change, t: t test statistic, *p*: *p*-value, Adj. *p*: Bonferroni-adjusted *p*-value.

| ID | Log2FC | t | *p* | Adj. *p* |
| --- | --- | --- | --- | --- |
| MATN2 | 0.647 | 4.235 | 0.002 | 0.212 |
| PGAM1 | 1.722 | 2.561 | 0.029 | 0.490 |
| THBS1 | -1.960 | -7.956 | 0.000 | 0.020 |
| FSTL3 | -1.619 | -4.730 | 0.001 | 0.129 |
| PSMD7 | -0.938 | -4.041 | 0.003 | 0.256 |
| LGALS3 | -0.446 | -3.268 | 0.009 | 0.425 |
| SYNCRIP | -0.470 | -3.184 | 0.010 | 0.425 |
| IGFBP6 | -0.527 | -3.018 | 0.013 | 0.425 |
| YES1 | -0.667 | -3.002 | 0.014 | 0.425 |
| MED1 | -0.412 | -2.946 | 0.015 | 0.425 |
| HNRNPAB | -0.462 | -2.939 | 0.015 | 0.425 |
| YWHAZ | -0.408 | -2.846 | 0.018 | 0.425 |
| PRDX6 | -0.436 | -2.825 | 0.019 | 0.425 |
| FGF1 | -0.745 | -2.824 | 0.019 | 0.425 |
| HSPA8 | -0.414 | -2.810 | 0.019 | 0.425 |
| YWHAB | -0.391 | -2.654 | 0.025 | 0.480 |
| PPIA | -0.411 | -2.593 | 0.028 | 0.490 |
| CCL2 | -0.369 | -2.551 | 0.030 | 0.490 |
| TNFRSF12A | -0.433 | -2.534 | 0.030 | 0.490 |
| TPM4 | -0.459 | -2.492 | 0.033 | 0.490 |
| ADSL | -0.354 | -2.473 | 0.034 | 0.490 |
| CDH3 | -0.355 | -2.363 | 0.041 | 0.490 |
| ABL1 | -0.310 | -2.339 | 0.042 | 0.490 |
| ADAMTS4 | -0.705 | -2.331 | 0.043 | 0.490 |
| ENG | -0.329 | -2.309 | 0.044 | 0.490 |

| ID | log2FC | t | *p* | Adj. *p* |
| --- | --- | --- | --- | --- |
| FSTL3 | -0.821 | -6.427 | 0.000 | 0.068 |
| CCDC80 | -0.800 | -4.595 | 0.002 | 0.155 |
| THBS1 | -0.665 | -6.943 | 0.000 | 0.068 |
| FN1 | -0.608 | -2.861 | 0.023 | 0.328 |
| DLL4 | -0.202 | -4.015 | 0.005 | 0.185 |
| NTN1 | -0.195 | -3.034 | 0.018 | 0.318 |
| C1QBP | -0.177 | -2.672 | 0.030 | 0.341 |
| CDH2 | -0.154 | -3.711 | 0.007 | 0.228 |
| BDNF | -0.151 | -2.431 | 0.044 | 0.364 |
| IGFBP6 | -0.145 | -2.551 | 0.036 | 0.342 |
| MAP2K3 | -0.095 | -2.879 | 0.022 | 0.324 |
| NAAA | 0.094 | 2.449 | 0.042 | 0.356 |
| MPO | 0.147 | 2.988 | 0.019 | 0.322 |
| GNS | 0.156 | 3.424 | 0.010 | 0.265 |
| HGF | 0.157 | 2.878 | 0.022 | 0.324 |
| CXCL12 | 0.172 | 2.714 | 0.028 | 0.341 |
| PIGR | 0.177 | 2.740 | 0.027 | 0.341 |
| B2M | 0.177 | 2.754 | 0.027 | 0.341 |
| NCR3 | 0.183 | 3.427 | 0.010 | 0.265 |
| GRN | 0.204 | 5.142 | 0.001 | 0.113 |
| NRP1 | 0.242 | 3.655 | 0.007 | 0.237 |
| AKR1A1 | 0.255 | 4.905 | 0.001 | 0.132 |
| FGR | 0.263 | 3.342 | 0.011 | 0.269 |
| SPARCL1 | 0.316 | 2.406 | 0.045 | 0.369 |
| SEMA5A | 0.388 | 5.442 | 0.001 | 0.104 |
| MATN2 | 0.407 | 3.438 | 0.010 | 0.265 |
| CCL21 | 0.425 | 3.649 | 0.007 | 0.237 |
| CCL5 | 0.461 | 5.288 | 0.001 | 0.106 |
| CXCL11 | 0.609 | 4.810 | 0.002 | 0.136 |
| BTK | 0.685 | 3.266 | 0.013 | 0.281 |

**Table S10** Overlap of significant genes and proteins detected in Lipopolysacharide(+) conditions. ID: gene or protein name, Log_2_FC: log_2_ fold change, t: t test statistic, *p*: *p*-value, Adj. *p*: Bonferroni-adjusted *p*-value.

**Table S11** Overlap of significant processes between genomics and proteomics. nes = normalized enrichment score; LPS = lipopolysaccharide.

| id | description | nes | *p* |
| --- | --- | --- | --- |
| LPS- |  |  |  |
| GO:0002768 | immune response-regulating cell surface receptor signaling pathway | 1.750 | 0.011 |
| GO:0071560 | cellular response to transforming growth factor beta stimulus | -1.419 | 0.011 |
| GO:0071559 | response to transforming growth factor beta | -1.489 | 0.004 |
| LPS+: |  |  |  |
| GO:0030595 | leukocyte chemotaxis | 1.658 | 0.005 |
| GO:0051251 | positive regulation of lymphocyte activation | 1.501 | 0.008 |
| GO:1903706 | regulation of hemopoiesis | 1.506 | 0.017 |
| GO:0002521 | leukocyte differentiation | 1.500 | 0.018 |
| GO:0050900 | leukocyte migration | 1.435 | 0.020 |
| GO:0051249 | regulation of lymphocyte activation | 1.418 | 0.023 |
| GO:0046649 | lymphocyte activation | 1.404 | 0.038 |
| GO:0060326 | cell chemotaxis | 1.322 | 0.044 |
| GO:0032944 | regulation of mononuclear cell proliferation | 1.366 | 0.049 |
| GO:0034329 | cell junction assembly | -1.566 | 0.022 |
